# Femoral neck shaft angle measurement on plain radiography: is standing or supine radiograph a reliable template for the contralateral femur?

**DOI:** 10.1186/s12891-022-06071-5

**Published:** 2022-12-14

**Authors:** Bassem Haddad, Mohammad Hamdan, Mohammad Al Nawaiseh, Osama Aldowekat, Mohammad Ali Alshrouf, Abdulrahman M. Karam, Muayad I. Azzam, Anas AR Altamimi, Muntaser Abu Shokor

**Affiliations:** 1 Department of Special Surgery, Division of Orthopedics, School of Medicine, The University of Jordan, Amman, Jordan; 2 The School of Medicine, The University of Jordan, Amman, 11942 Jordan; 3 Department of Special Surgery Faculty of Medicine, The Hashemite University, Zarqa, Jordan

**Keywords:** Femoral neck shaft angle, NSA, Neck shaft angle, Proximal femur, Fracture, Femoral neck fractures, Radiographs

## Abstract

**Introduction:**

Neck-shaft angle (NSA) is of paramount importance to orthopedic surgeons due to its implications for various pathologies of the hip and femur. The primary aim of the study was to establish if NSA measurement may be affected by imaging position (standing and supine) and provide evidence regarding whether the contralateral NSA can be used as a template. The secondary aim was to determine a reference value and precisely understand the effects of sex on NSA measurement.

**Materials and methods:**

We measured bilateral NSA in a retrospective study of 200 standing and 200 supine anteroposterior pelvis radiographs that met the inclusion criteria, while paying special attention to bilateral hip symmetry. The overall inter-rater reliability was 0.688 (CI 0.128–0.851). Matching was performed according to sex (exact matching) and age. Paired t-test, Pearson correlation coefficient, and independent sample t-test were used (*p* < 0.01).

**Results:**

A total of 400 pairs of femoral necks were reviewed, comprising of 200 males and 200 females. In the upright radiograph, the overall mean NSA was 131.21° ± 4.72°. There was no significant difference between right and left femur NSA among the patients (*p* = 0.95). On both sides, male NSA was higher than female NSA (*p* < 0.001). In supine radiograph, the overall mean NSA for the supine position was 133.06° ± 5.71°. There was a significant difference between NSA of the right and left femur among the patients in the supine position (*p* < 0.001). On supine radiographs there was no statistically significant difference between male and female NSA (*p* = 0.85).

**Conclusion:**

Our findings indicated no significant variability in upright radiographs between the right and left NSA. In contrast, significant asymmetry between the right and left NSA was found in the supine radiographs. However, this study does not provide definitive clinical evidence, and further clinical-oriented research is required.

**Level of evidence:**

Level III; retrospective comparative study.

**Supplementary Information:**

The online version contains supplementary material available at 10.1186/s12891-022-06071-5.

## Introduction

Femoral neck-shaft angle (NSA), also known as the caput-collum-diaphyseal angle, is the intersection between the proximal femoral shaft axis and the femoral neck axis. It plays a role in diagnosis or management of several hip and femur problems, such as osteoarthritis, hip fractures, greater trochanteric pain syndrome, cerebral palsy, and femoroacetabular impingement [1–5]. As a result of its critical significance, preoperative imaging techniques and measurement methods must be accurate, as they may affect the reliability and measurement accuracy that may render patients unable to achieve their maximal potential functional capacity. Especially in cases of significant discrepancies, the surgeon’s decision may be affected as a result. Therefore, anteroposterior radiographs were standardized in order to measure the NSA and minimize the protectional errors due to hip rotation [6–8].

Neck-shaft angle is considered one of the most commonly measured indicators of hip anatomy [9]. A multi-national analysis of 8271 femora found that human neck-shaft angles varied from 120° to 140°, with a mean of 126.4° [10]. A recent study showed a mean value of 131.3° [11]. These values are subject to a wide range of discrepancies, as it was demonstrated that age varies inversely with the angle [12]. However, opposite findings have also been reported [13]. Moreover, some studies revealed greater neck-shaft angles in females [12], while others found higher values in males [13]. In addition, Gilligan et al. showed a 1.3° significant difference between both sides [10]. Nevertheless, other studies failed to find significant side asymmetry [14, 15].

It remains a subject of debate among orthopedic surgeons globally, since there is a limited literature on NSA measurements and there is no established measurement protocol to measure NSA. The aim of the study is to determine side-to-side variability, to determine whether and to what extent imaging position, including standing and supine, can affect NSA measurement, to provide reference values, and to provide perspective on possible sex-based differences. This will guide orthopedic surgeons during the management of several pathologies around the femoral neck-shaft angle. We hypothesized that there is no significant difference between the bilateral NSA and that the contralateral femur can be used as a template during proximal femur fracture pre-operative planning and other indications.

## Methodology

### Study design

This is a multi-center retrospective study, which involved measuring the NSA using anteroposterior pelvic radiographs of adult patients. A sample size of 400 anteroposterior view of the pelvis were selected, showing both hip joints and a sufficient part of the upper femur. Ethical approval was granted by the appropriate Institutional Review Board (IRB) of the Jordan University Hospital. This study was conducted in accordance with the Declaration of Helsinki.

The study investigated four associations. Firstly, determine the side-to-side variability to determine the reliability of using the contralateral hip as a template in order to restore the patient's original morphology. Secondly, assess NSA variability between upright and supine pelvic anteroposterior radiographs of adult hips with no pathologies. Thirdly, provide a reference value of the neck-shaft angle and investigate the effects of sex on NSA.

### Study sample and population

**Fig. 1:**
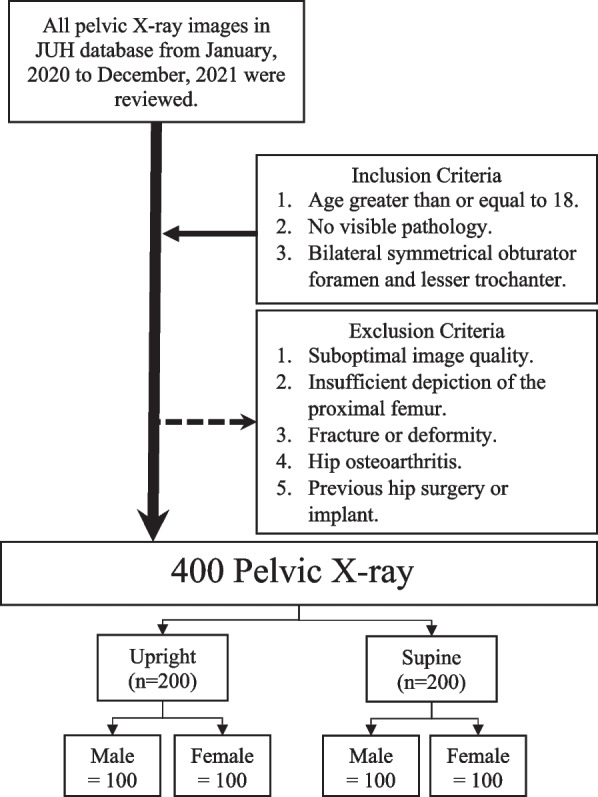
Summary of the study design

Patients aged 18 years or older who did not have any visible hip pathology and had bilaterally symmetrical obturator foramen and lesser trochanters on the X-ray were included in the study. Exclusion criteria included suboptimal image quality, insufficient depiction of the proximal femur, fracture, deformity, hip osteoarthritis, or previous hip surgery or implant. Upright and supine X-rays were retrieved by 6^th^ year trained medical students, while paying special attention to bilateral hip symmetry by ensuring that the obturator foramina and the lesser trochanter were symmetrical bilaterally. Images were reviewed by two senior orthopedics residents before being included. Figure 1 demonstrates the data collection process in detail.

### Data collection

The data were retrieved from radiological databases at a 600-bed tertiary care teaching hospital and a 250-bed tertiary care hospital. Images over two years, between January 2020 and December 2021, were reviewed to ensure they met all of the inclusion and exclusion criteria. Matching was performed according to sex (exact matching) and age.

The final selected list was reviewed again to ensure the obtained images satisfied the inclusion and exclusion criteria by two independent examiners specialized in orthopedic surgery, then used a goniometer on the picture archiving and communication system (PACS) to measure the neck-shaft angle on each of the 800 femoral necks to measure the NSA.

### NSA measurement

**Fig. 2:**
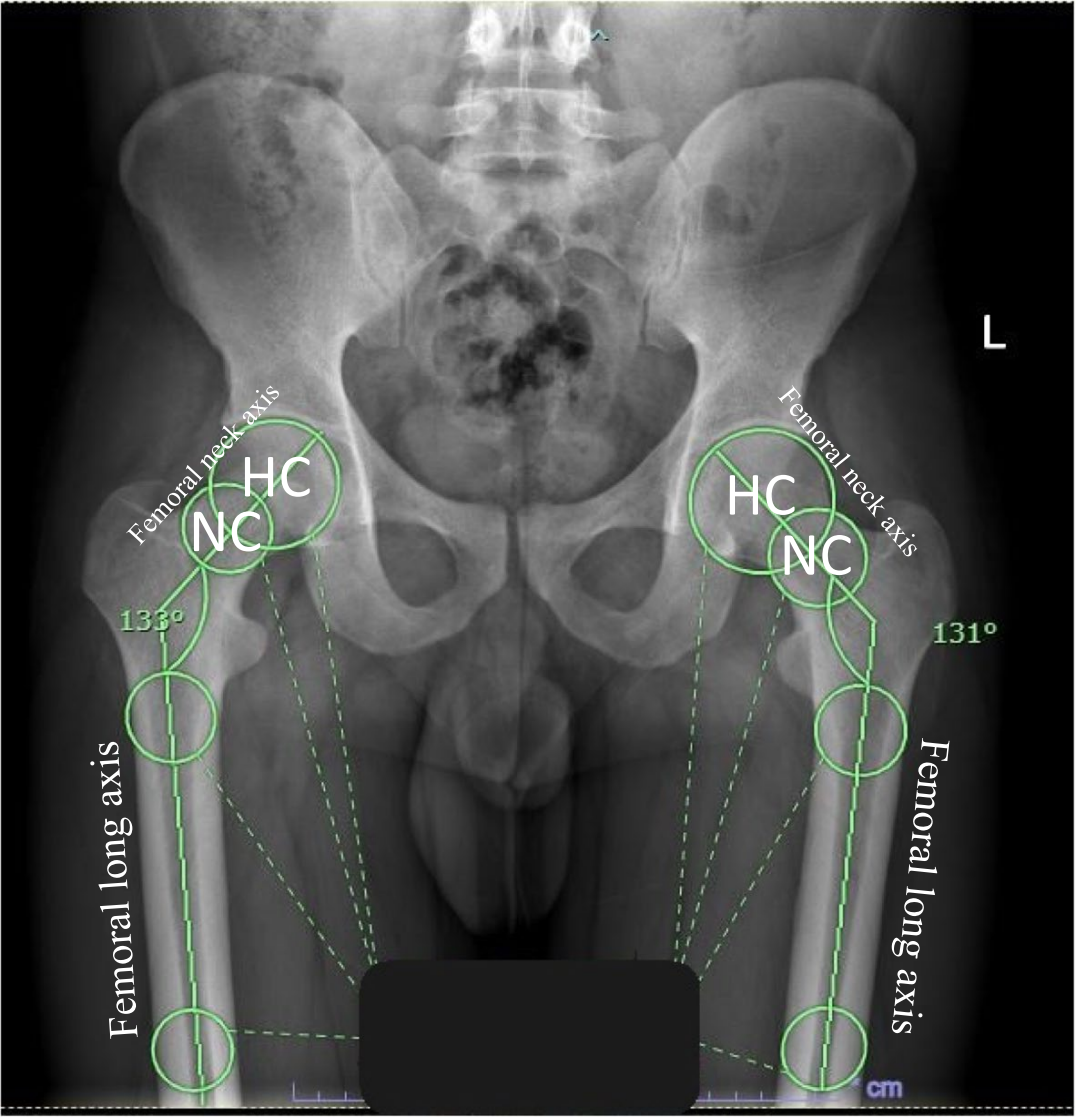
The measurement method of neck-shaft angle on upright pelvis plain radiograph. HC, femoral head center; NC, femoral neck center

A well-defined protocol for measuring NSA bilaterally was determined prior to starting. The NSA was measured by drawing a line between the femoral neck axis and the femoral long axis. The measurements were based on the technique proposed by Boese et al. [9], which provided the following definitions: (1) The femoral neck axis was defined as the line connecting the femoral head center (HC) and center of the femoral neck (NC); (2) the NC was defined as the center between the cutting points of a circle centered on the HC and the lower and upper margin of the femoral neck; (3) the femoral long axis was defined using the two most common markers, the proximal or distal center of the femoral shaft. Figure 2 illustrates the measurement technique of NSA on an upright pelvis plain radiograph.

### Statistical analysis

SPSS version 28.0 (Chicago, USA) was used for statistical analysis. Variability analysis in the form of mean (standard deviation), median, and range was used to describe the data. The presented measurements in the study are the mean value of measurements that was calculated the first time by the two examiners. The difference in means between the NSA of the right and left femur was compared using a paired t-test. Correlations analysis between sex reference values, left and right NSA were measured using the Pearson correlation coefficient (r). Independent sample t-test was used to compare the mean NSA by sex. Box plot was used to demonstrate the outliers. After two months, the same two reviewers were asked to repeat the measurement of 40 randomly selected X-rays (80 hips) again to calculate the intra-rater reliability. Intraclass correlation coefficients (ICCs) for inter-rater reliability were conducted to compare the measurements for each parameter of the first and second measures as per the guidelines by [16]. The analyses intra-rater reliability for NSA measurement on upright and supine radiographs was determined using ICC and a confidence interval (CI) of 95%. A *p*-value < 0.01 was considered statistically significant.

## Results

A total of 800 femoral neck-shaft angles were analysed from 400 patients’ radiographs, divided into 200 upright radiographs and 200 supine radiographs. The male to female ratio for the study participants was 1:1. Mean age was similar among all groups, and there was no statistically significant mean difference in age between the sex in the upright (46.36 ± 11.74 for females and 45.84 ± 16.74 for males) or supine (42.78 ± 15.86 for females and 42.64 ± 16.21 for males) positions (*p* = 0.800, 0.951, respectively) (Table 1).

**Table 1:** Demographics of the upright and supine radiographs

	**Upright**		**Supine**	
	**Count (%)**	**Mean ± SD**	**Count (%)**	**Mean ± SD**
Sex				
Female	100 (50%)		100 (50%)	
Male	100 (50%)		100 (50%)	
Age (years)				
Female		46.36 ± 11.74		42.78 ± 15.86
Male		45.84 ± 16.74		42.64 ± 16.21

*SD* Standard deviation

All NSA measurements were carried out twice by two specialized orthopedic members of the research team separately. The overall inter-rater reliability for both left and right femora was 0.688 (CI 0.128–0.851) (Table 2). The intra-rater reliability rates for the two reviewers were 0.898 (95% CI 0.840–0.934) and 0.858 (95% CI 0.736–0.918). A significant difference was found between the mean of the upright radiograph NSA and the supine radiograph NSA (*p* < 0.001), with a mean difference of 1.85° higher in the supine radiograph (95% CI 2.88 to 8.23).

**Table 2:** Inter-rater reliability with 95% confidence interval for right and left for both upright and supine

Variables	Inter-rater reliability (95% CI)
Upright, right	0.797 (CI 0.691–0.861)
Upright, left	0.590 (CI 0.174–0.769)
Supine, right	0.758 (CI -0.078–0.912)
Supine, left	0.603 (CI -0.211–0.855)
Overall	0.688 (CI 0.128–0.851)

*CI* Confidence Interval

### Measuring neck-shaft angle on pelvic radiographs in the upright position

In the upright position, the mean age of all 200 patients who underwent upright radiographs was 46.10 ± 14.42 years, with age ranging from 18 to 81 years; females had a mean age of 46.36 ± 11.74 years (range, 18 to 66) and males of 45.84 ± 16.74 years (range, 19 to 81).

Overall, the mean NSA in the upright position was 131.21° ± 4.72°. There was no significant difference between the right and left femur NSA among the patients in the upright position (*p* = 0.95). In addition, in the overall assessment, there was a strong statistically significant relationship between the right and left NSA in the upright pelvic radiographs (*r* = 0.789, *p* < 0.001).

The NSA in males ranged from 119.5° to 145° with a mean of 132.29° ± 4.38°. There was no statistical difference between the mean NSA value for males 132.31° ± 4.71° on the right side and 132.26° ± 4.54° on the left side (*p* = 0.84). Females had NSA values ranging from 116.5° to 145.5° with a mean value of 130.13° ± 4.83°. No statistical difference was found between the right side's mean value of 130.51° ± 5.41°, and the left side's mean value of 129.75° ± 4.89° (*p* = 0.035). Table 3 shows the mean values of NSA in detail according to sex and side.

**Table 3:** Demographic and neck-shaft angle characteristics

	**NSA Range**	**Mean ± SD (Right)**	**Mean ± SD (Left)**	**Paired t-test, *p* Value**	**Pearson Correlation Coefficient (r), *p* Value**	**Mean ± SD (Right and left)**
**Upright**						
Male	119.5–145	132.31 ± 4.71	132.26 ± 4.54	0.84	0.789, < 0.001	132.29 ± 4.38
Female	116.5–145.5	130.51 ± 5.41	129.75 ± 4.89	0.035	0.763, < 0.001	130.13 ± 4.83
Overall	116.5–145.5	131.41 ± 5.14	131.00 ± 4.87	0.95	0.789, < 0.001	131.21 ± 4.72
**Supine**						
Male	120.5–152.5	134.23 ± 6.16	132.05 ± 5.53	< 0.001	0.796, < 0.001	133.14 ± 5.54
Female	117.5–148.5	133.97 ± 6.51	131.99 ± 5.83	< 0.001	0.830, < 0.001	132.98 ± 5.90
Overall	117.5–152.5	134.10 ± 6.32	132.03 ± 5.67	< 0.001	0.814, < 0.001	133.06 ± 5.71

*SD* Standard deviation, *NSA* Neck-shaft angle; *p* Value indicates the mean difference and correlation between the NSA mean of the right and left sides

In regard to the NSA measurement difference between females and males in the supine position, the mean value of the NSA in males was significantly higher (*p* < 0.001).

The majority of patients (87.5%) had a difference less than 5° in bilateral NSA, and only three had a difference greater than 10°, and greatest difference was 11°. Almost half (46.5%) of the patients fell within the 130°-135° range (Table 4).

**Table 4:** Intra-patient variation in neck-shaft angle

Difference in NSA	Total n (%)	Female n (%)	Male n (%)
**Upright**			
< 5°	175 (87.5)	84 (84)	91 (91)
5–10°	22 (11)	14 (14)	8 (8)
> 10°	3 (1.5)	2 (2)	1 (1)
**Supine**			
< 5°	153 (76.5)	76 (76)	77 (77)
5–10°	43 (21.5)	22 (22)	21 (21)
> 10°	4 (2)	2 (2)	2 (2)

*NSA* Neck-shaft angle

### Measuring neck-shaft angle on pelvic radiographs in the supine position

In the supine position, the mean age of the 200 patients was 42.71 ± 16 years, with an age range of 18 to 78 years; females had a mean age of 42.78 ± 15.86 years (range, 18 to 78) and males of 42.64 ± 16.21 years (range, 18 to 77).

Overall, the mean NSA in the supine position was 133.06° ± 5.71°. In contrast to the upright radiography, there was a significant difference between NSA of right and left femur among the patients in the supine position (*p* < 0.001). However, similar to the upright radiography, there was a strong statistically significant relationship between the right and left NSA in the supine pelvic radiographs (*r* = 0.814, *p* < 0.001).

The NSA of males ranged from 120.5° to 152.5°, with a mean of 133.14° ± 5.54°. There was a statistical difference between the mean NSA value for males 134.23° ± 6.16° on the right side and 132.05° ± 5.53° on the left side (*p* < 0.001). Females had NSA values ranging from 117.5° to 148.5° with a mean value of 132.98° ± 5.90°. Also, there was a statistically significant difference between the right side's mean value of 133.97° ± 6.51° and the left side's mean value 131.99° ± 5.83° (*p* < 0.001).

In regard to the NSA measurement difference between females and males in the supine position, there was no statistically significant difference in the mean values of the NSA between the females and males (*p* = 0.85).

**Fig. 3:**
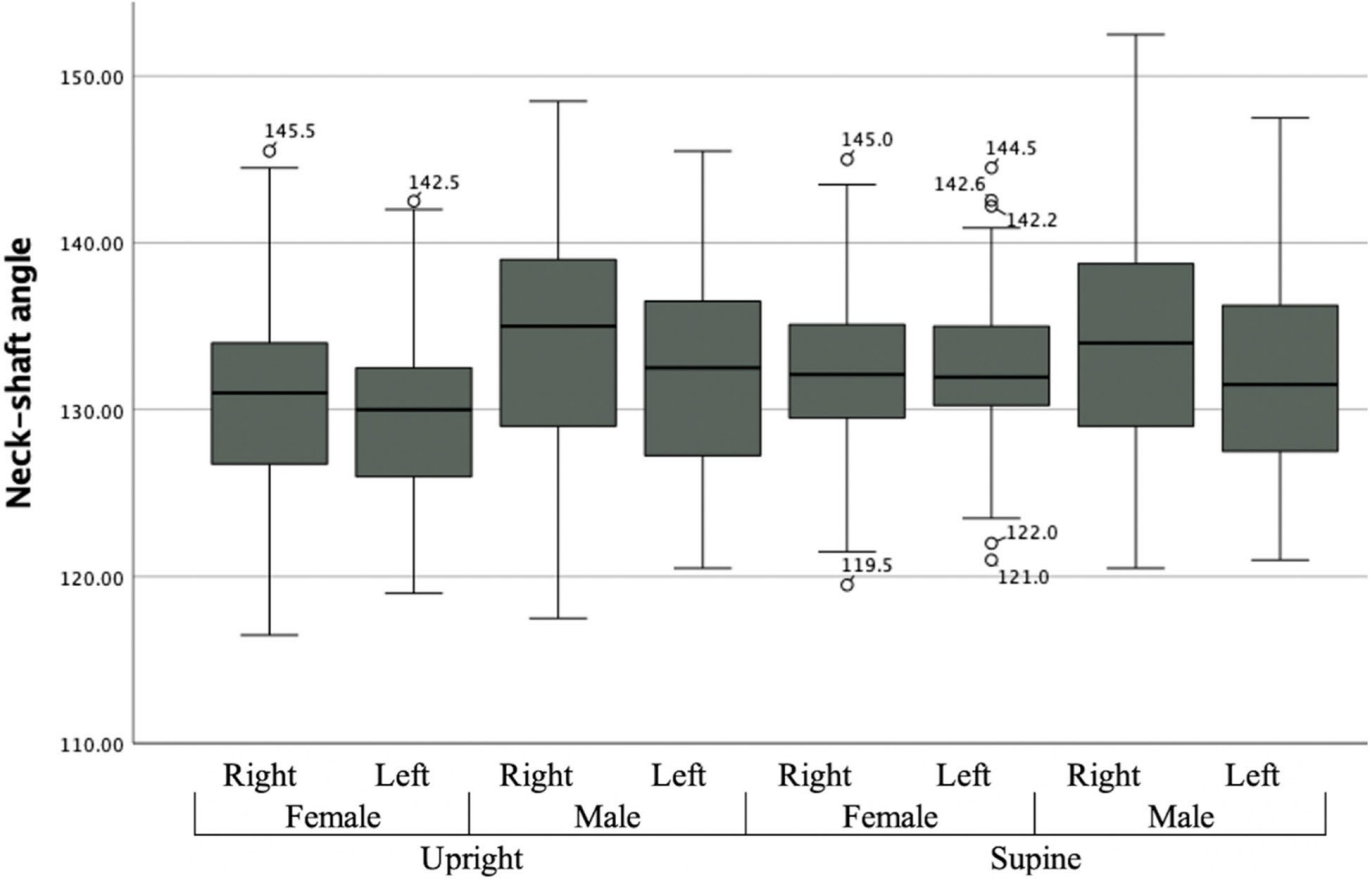
Box Plot showing the neck-shaft angle measurements on the upright and supine imaging for females and males

The majority of patients (76.5%) had a difference less than 5° in bilateral NSA, and only four had a difference greater than 10°, and greatest difference was 11.5° (Table 4). Figure 3 demonstrates the Box Plot showing the neck-shaft angle measurements on the upright and supine imaging for females and males.

## Discussion

The aim of this study was to test for side-to-side variability, to determine the effect of patient positioning on the NSA using both anteroposterior supine and upright pelvic radiographs, and to provide reference NSA values, as well as studying differences in terms of sex. According to our findings, the mean NSA on the upright radiographs was found to be 131.21° ± 4.72°, and on the supine position the mean NSA was 133.06° ± 5.71°. Our results showed no significant side-to-side variability between the left and right femur on upright X-ray; however, a significant difference was found with regards to the supine position. Our study also showed a significant difference in the mean values of the NSA between females and males only in the upright positions. Additionally, a significant difference was found in the NSA between the upright and supine position. Overall, the study had an intra-rater ICC of 0.898 and 0.858 for the two reviewers.

Femoral neck angle is known to vary during growth, between geographical locations, and across temporal periods. The cause of these differences between distinct regions and populations is hypothesized to be either as a result of differences in activity levels or a consequence of climate-induced body proportions [17]. For our sample, the mean NSA was found to be 131.21° ± 4.72° in the upright position and 133.06° ± 5.71° for supine radiographs. In a systematic review including 26 publications reporting the measurement of the NSA on conventional radiograph, the mean NSA of healthy adults (5,089 hips) was 128.8° [9]. In the systematic review, the NSA ranged from 123° to 137.3°, showing significant variation between the studies. The variability could be the result of the geographical differences as well as the different methods used to measure the NSA. Similar studies done on NSA using other radiological modalities, such as computed tomography and dual-energy X-ray absorptiometry (DEXA) scans, have described a similar range for NSA values [18–20]. In our sample, the NSA value was found to be higher than the mean of most studies found in the literature. Anatomical studies done on femoral neck angle measurement have shown a significant increase in mean neck-shaft angles in populations with a sedentary lifestyle [21], which may contribute to the higher NSA as numerous studies have described many population to have a public health problem of sedentary lifestyle and higher obesity rates [22, 23]. The pelvic bony geometry is complex and known differences exist between both sexes. Our study also showed a significant difference in the mean values of the NSA between females and males in upright positions but not on the supine position. This is similar to the finding of Chiu et al. that amongst the Malaysian population, females had a significantly higher NSA than males [24]. Nevertheless, various studies have shown no differences in NSA between males and females [25, 26].

Femoral neck angle measurement has various clinical implications in adults, one potential implication is to aid in determining NSA after an injury. This is of importance in the fixation of unstable hip fractures as having a nail angle less than the native NSA leads to more varus reductions and fracture displacement [27]. Some studies have hypothesized that due to the dominance of one leg over another, bone lengths and angles may be affected [28]. Our results showed no significant side-to-side variability between the left and right femur on upright X-rays; however, a significant difference was observed in the supine position. In a study done by Rogers et al. on 203 patients to check for side-to-side variability of the NSA using upright anteroposterior pelvis radiographs, no significant variability between the two angles was found [29]. Similarly, a study done in India on 110 patients using supine anteroposterior pelvis radiographs concluded that the NSA angle of the contralateral femur can be used as a template during repair [25]. Future randomized control trials comparing the outcome of using the NSA of the contralateral femur versus other methods during surgery would provide more conclusive evidence.

Our study had an intra-rater ICC of 0.898 (95% CI 0.840–0.934) and 0.858 (95% CI 0.736–0.918) of the two reviewers and an overall inter-rater reliability of 0.688 (CI 0.128–0.851). Mast et al. in their study on 20 radiological images, found the intra-rater reliability of 0.94 for the first observer and 0.95 for the second observer and inter-rater reliability of NSA to be 0.58 [30], while Nelitz et al. reported an intra-rater reliability range between 0.76 to 0.90 and inter-rater reliability range between 0.72 to 0.89 [31]. Bouttier et al. has reported the same high intra-rater reliability of 0.90 for both observers and inter-rater reliability of 0.83 [32]. Nevertheless, other studies have shown higher ICC on computed tomography, such that one study done by Boese et al. reported an a intra-rater reliability of 0.995 and inter-rater reliability of 0.914 [33]. The authors attribute this higher intra-rater and inter-rater reliability to the negation of the femoral rotation effect in CT scans and clearly defined measurement protocol. This suggests that the ICC for the NSA has moderate to good reliability according to the cut-off values reported by Koo and Li, and the reliability can be increased when using a CT scan compared to the X-ray images [16].

To date, there is no established method for measuring the NSA. While some studies have described methods that utilize pelvic radiographs [2, 34, 35], other studies have described methods that require imaging the whole femur [36, 37]. Furthermore, some studies have found a difference between non-corrected and rotation-corrected measurements of the NSA, such that in a systematic review done by Boese et al., with positional correction the NSA was found to be 128.8° versus 131.6° in the subgroup without correction [9]. To add to this, our study compared NSA in an upright versus supine position, such that a significant difference was found between the two X-ray radiographic patient positioning. According to our search, no other study has compared the NSA between the two positions. Taking pelvic radiographs in the upright position may allow for a more accurate assessment of the NSA when the pelvis is influenced by weight-bearing. Also, the upright position may limit the patient from unknowingly internally or externally rotating the femur during the imaging process. However, taking an upright image may be impossible when a patient has a hip fracture. Various studies have also suggested other imaging modalities to measure NSA [18–20, 38].

In a study that compared radiographic measurements on standing and supine pelvic radiographs, it was found that standing pelvic radiographs resulted in lower lateral center edge angle and acetabular depth measurements, a lower likelihood of a positive crossover sign or ischial spine sign, and a higher acetabular inclination. Accordingly, they suggested using standing anteroposterior pelvic radiographs to obtain the most precise pelvic radiographic parameters [39]. Another study compared supine and standing pelvis radiographs in the evaluation of pincer-type femoroacetabular impingement, found that in the standing position there was a decrease in the incidence and amount of the ischial spine sign and crossover sign and a small increase in inclination; therefore, they recommended the use of standing pelvis radiographs in nonarthritic hip pain [40]. Trying to explain our results in supine radiographs, pelvis X-rays taken in a supine position might not have been absolutely symmetrical, allowing for a significant difference in NSA measurements. This could be the case because, in a standing posture, it is much simpler to manage the little variations in rotation in both hips to create symmetrical hips; however, in the supine position, it is harder to control the symmetry between the right and left femur as well as the rotation. We suggest that radiographs to be taking in standing position whenever possible since it is more clinically relevant because the pelvis is in a more functional position. Another reason might be the effect of lower limb dominance mentioned earlier, which is usually the right side [28]. However, lower limb dominance was not addressed in this study, so this remains a plausible but hypothetical explanation.

This study's main strength is that it presents a comprehensive assessment of the NSA measurement on radiographic images using a well-defined measurement method that enables the reproducibility of the study for future comparison. In addition, this is the first study to directly compare supine and standing plain pelvic radiographs with regards to femoral neck-shaft angle. The study’s limitations are mainly a result of the retrospective design, which caused us to rely solely on the patients’ records to exclude any deformities or previous trauma that may have affected the NSA. Secondly, the study sample was limited due to numerous radiographical images being excluded as a result of poor image quality and asymmetry.

## Conclusion

To conclude, our study provides preliminary evidence that supports the use of the femoral neck-shaft angle measurement on standing pelvic X-rays of the contralateral femur to aid in determining the angle of the pathological side. Taking into consideration the results of this study and the fact that the pelvis is in a more functional position, we recommend the use of standing radiographs to measure the NSA whenever possible. Furthermore, the surgeon must be aware of the possible difference in the NSA measurement based on whether the radiographs are obtained standing or supine position. In addition, we recommend that an established method to measure NSA be determined to limit the variation of NSA values currently found in the literature.

## Supplementary Information

**Additional file 1.**

## Data Availability

The data from the present research that were utilized and analyzed are available online as a supplementary material.

## References

[1] Shoji T, Yamasaki T, Izumi S (2016). The influence of stem offset and neck shaft angles on the range of motion in total hip arthroplasty. Int Orthop.

[2] Doherty M, Courtney P, Doherty S (2008). Nonspherical femoral head shape (pistol grip deformity), neck shaft angle, and risk of hip osteoarthritis: a case-control study. Arthritis Rheum.

[3] Ripamonti C, Lisi L, Avella M (2014). Femoral neck shaft angle width is associated with hip-fracture risk in males but not independently of femoral neck bone density. Br J Radiol.

[4] Fearon A, Stephens S, Cook J (2012). The relationship of femoral neck shaft angle and adiposity to greater trochanteric pain syndrome in women. A case control morphology and anthropometric study. Br J Sports Med.

[5] Bouma H, Hogervorst T, Audenaert E, van Kampen P (2015). Combining femoral and acetabular parameters in femoroacetabular impingement: the omega surface. Med Biol Eng Comput.

[6] Kay RM, Jaki KA, Skaggs DL (2000). The effect of femoral rotation on the projected femoral neck-shaft angle. J Pediatr Orthop.

[7] Lechler P, Frink M, Gulati A (2014). The influence of hip rotation on femoral offset in plain radiographs. Acta Orthop.

[8] Merle C, Waldstein W, Pegg E (2012). Femoral offset is underestimated on anteroposterior radiographs of the pelvis but accurately assessed on anteroposterior radiographs of the hip. J Bone Joint Surg Br.

[9] Boese CK, Dargel J, Oppermann J (2016). The femoral neck-shaft angle on plain radiographs: a systematic review. Skeletal Radiol.

[10] Gilligan I, Chandraphak S, Mahakkanukrauh P (2013). Femoral neck-shaft angle in humans: variation relating to climate, clothing, lifestyle, sex, age and side. J Anat.

[11] Altubasi I, Hamzeh H, Madi M (2020). Measurement of Neck-Shaft Angle Using CT Scout View in Healthy Jordanian Adults - A Reliability and Agreement Study. J Adv Med Med Res.

[12] Fischer CS, Kühn J-P, Völzke H (2020). The neck-shaft angle: an update on reference values and associated factors. Acta Orthop.

[13] Elbuken F, Baykara M, Ozturk C (2012). Standardisation of the neck-shaft angle and measurement of age-, gender- and BMI-related changes in the femoral neck using DXA. Singapore Med J.

[14] Boese CK, Frink M, Jostmeier J (2016). The Modified Femoral Neck-Shaft Angle: Age- and Sex-Dependent Reference Values and Reliability Analysis. BioMed Res Int.

[15] Jiang N, Peng L, Al-Qwbani M (2015). Femoral version, neck-shaft angle, and acetabular anteversion in Chinese Han population: a retrospective analysis of 466 healthy adults. Medicine (Baltimore).

[16] Koo TK, Li MY (2016). A Guideline of Selecting and Reporting Intraclass Correlation Coefficients for Reliability Research. J Chiropr Med.

[17] Child SL, Cowgill LW (2017). Femoral neck-shaft angle and climate-induced body proportions. Am J Phys Anthropol.

[18] Gnudi S, Sitta E, Pignotti E (2012). Prediction of incident hip fracture by femoral neck bone mineral density and neck-shaft angle: a 5-year longitudinal study in post-menopausal females. Br J Radiol.

[19] Wright D, Whyne C, Hardisty M (2011). Functional and anatomic orientation of the femoral head. Clin Orthop.

[20] Buller LT, Rosneck J, Monaco FM (2011). Relationship Between Proximal Femoral and Acetabular Alignment in Normal Hip Joints Using 3-Dimensional Computed Tomography. Am J Sports Med.

[21] Anderson JY, Trinkaus E (1998). Patterns of sexual, bilateral and interpopulational variation in human femoral neck-shaft angles. J Anat.

[22] Sharkas GF, Saheb T, Arqoub K, Haddadin R (2016). Sedentary lifestyle among adults in Jordan. Fam Med Community Health.

[23] Khader Y, Batieha A, Ajlouni H (2008). Obesity in Jordan: prevalence, associated factors, comorbidities, and change in prevalence over ten years. Metab Syndr Relat Disord.

[24] Chiu CK, Chan CYW, Singh VA (2009). Is the femoral neck geometry adequate for placement of the proximal femoral nail in the Malaysian population? A review of 100 cases. Med J Malaysia.

[25] Pathak SK, Maheshwari P, Ughareja P (2016). Evaluation of femoral neck shaft angle on plain radiographs and its clinical implications. Int J Res Orthop.

[26] Reikerås O, Høiseth A (1982). Femoral neck angles in osteoarthritis of the hip. Acta Orthop Scand.

[27] Parry JA, Barrett I, Schoch B (2018). Does the Angle of the Nail Matter for Pertrochanteric Fracture Reduction? Matching Nail Angle and Native Neck-Shaft Angle. J Orthop Trauma.

[28] Chhibber SR, Singh I (1970). Asymmetry in muscle weight and one-sided dominance in the human lower limbs. J Anat.

[29] Rogers MJ, King TL, Kim J, et al. Femoral Neck Shaft Angle and Management of Proximal Femur Fractures: Is the Contralateral Femur a Reliable Template?. J Orthop Trauma. 2021;35:529–34. 10.1097/BOT.0000000000002069 PMC10506416 33813545

[30] Mast NH, Impellizzeri F, Keller S, Leunig M (2011). Reliability and agreement of measures used in radiographic evaluation of the adult hip. Clin Orthop.

[31] Nelitz M, Guenther KP, Gunkel S, Puhl W (1999). Reliability of radiological measurements in the assessment of hip dysplasia in adults. Br J Radiol.

[32] Bouttier R, Morvan J, Mazieres B (2013). Reproducibility of radiographic hip measurements in adults. Joint Bone Spine.

[33] Boese CK, Jostmeier J, Oppermann J (2016). The neck shaft angle: CT reference values of 800 adult hips. Skeletal Radiol.

[34] Lequesne M, Malghem J, Dion E (2004). The normal hip joint space: variations in width, shape, and architecture on 223 pelvic radiographs. Ann Rheum Dis.

[35] Moore RJ, Fazzalari NL, Manthey BA, Vernon-Roberts B (1994). The relationship between head-neck-shaft angle, calcar width, articular cartilage thickness and bone volume in arthrosis of the hip. Br J Rheumatol.

[36] Reikerås O, Høiseth A, Reigstad A, Fönstelien E (1982). Femoral neck angles: a specimen study with special regard to bilateral differences. Acta Orthop Scand.

[37] Rubin PJ, Leyvraz PF, Aubaniac JM (1992). The morphology of the proximal femur. A three-dimensional radiographic analysis. J Bone Joint Surg Br.

[38] Sangeux M, Pascoe J, Graham HK (2015). Three-dimensional measurement of femoral neck anteversion and neck shaft angle. J Comput Assist Tomogr.

[39] Spiker AM, Graf RM, Duminie SP (2020). Differences in radiographic measurements on standing versus supine pelvic radiographs. Orthop J Sports Med.

[40] Jackson TJ, Estess AA, Adamson GJ (2016). Supine and standing AP pelvis radiographs in the evaluation of pincer femoroacetabular impingement. Clin Orthop.

